# Construction of *Mycobacterium tuberculosis cdd* knockout and evaluation of invasion and growth in macrophages

**DOI:** 10.1590/0074-02760170105

**Published:** 2017-11

**Authors:** Anne Drumond Villela, Valnês S Rodrigues-Junior, Antônio Frederico Michel Pinto, Virgínia Carla de Almeida Falcão, Zilpa Adriana Sánchez-Quitian, Paula Eichler, Cristiano Valim Bizarro, Luiz Augusto Basso, Diógenes Santiago Santos

**Affiliations:** 1 Pontifícia Universidade Católica do Rio Grande do Sul, Centro de Pesquisas em Biologia Molecular e Funcional, Instituto Nacional de Ciência e Tecnologia em Tuberculose, Porto Alegre, RS, Brasil; 2 Pontifícia Universidade Católica do Rio Grande do Sul, Programa de Pós-Graduação em Biologia Celular e Molecular, Porto Alegre, RS, Brasil; 3 Pontifícia Universidade Católica do Rio Grande do Sul, Programa de Pós-Graduação em Medicina e Ciências da Saúde, Porto Alegre, RS, Brasil

**Keywords:** Mycobacterium tuberculosis, *cdd* gene, cytidine deaminase

## Abstract

Cytidine deaminase (MtCDA), encoded by *cdd* gene (Rv3315c), is the only enzyme identified in nucleotide biosynthesis pathway of *Mycobacterium tuberculosis* that is able to recycle cytidine and deoxycytidine. An *M. tuberculosis* knockout strain for *cdd* gene was obtained by allelic replacement. Evaluation of mRNA expression validated *cdd* deletion and showed the absence of polar effect. MudPIT LC-MS/MS data indicated thymidine phosphorylase expression was decreased in knockout and complemented strains. The *cdd* disruption does not affect *M. tuberculosis* growth both in Mid- dlebrook 7H9 and in RAW 264.7 cells, which indicates that *cdd* is not important for macrophage invasion and virulence.

Tuberculosis is an infectious disease caused mainly by *Mycobacterium tuberculosis.* The World Health Organization estimated that 10.4 million people developed tuberculosis in 2015, resulting in 1.8 million deaths (WHO 2016). Although an efficient chemotherapy exists, affordable, short, effective and well-tolerated treatments for coadministration with anti-HIV agents, latent, drug-susceptible and drug-resistant tuberculosis are still needed to decrease the global incidence of the disease (Mdluli et al. 2015). The first step towards the search for novel therapeutic strategies is to better understand important metabolic pathways of the pathogen. Pyrimidine biosynthesis pathway provides pyrimidine nucleotides that are essential components of many biomolecules. Pyrimidine nucleotides in *M. tuberculosis* may be synthetised *de novo* from simple precursors, or may be obtained by the salvage pathway from preformed pyrimidine bases and nucleosides (Warner et al. 2014). While the *de novo* pathway is a high energy demanding process, the salvage pathway might be preferentially utilised under restricted energy availability (Villela et al. 2011).

Cytidine deaminase (MtCDA, EC 3.5.4.5) is encoded by *cdd* gene (Rv3315c, Gene ID: 887975), and is within *cdd-add* operon with *deoA* (Rv3314c, thymidine phos-phorylase, MtTP, EC 2.4.2.4) and *add* (Rv3313c, adenosine deaminase, MtAD, EC 3.5.4.4) genes (Roback et al. 2007). The *cdd* gene was predicted to be non-essential by Himar1-based transposon mutagenesis in H37Rv (Sassetti et al. 2003, Griffin et al. 2011). MtCDA is an important enzyme from the pyrimidine salvage pathway in *M. tuberculosis* that recycles cytidine and 2’-deoxycytidine for uridine and 2’-deoxyuridine synthesis, respectively (Sánchez-Quitian et al. 2010), and is the only enzyme identified by sequence homology in nucleotide biosynthesis pathway of *M. tuberculosis* that is able to rescue cytidine and deoxycytidine (Villela et al. 2011). Deoxyuridine can be converted to uracil by pyrimidine nucleoside phosphorylase (EC 2.4.2.4, *deoA,* Rv3314c) enzyme. Then, uracil phosphoribosyltransferase (EC 2.4.2.9, *upp,* Rv3309c) enzyme catalyses the conversion of uracil to uridine monophosphate (UMP), which is the precursor of all pyrimidine nucleotides (Villela et al. 2011). Uridine nucleosidase (EC 3.2.2.3) and uridine phosphorylase (EC 2.4.2.3) that convert uridine into uracil, and uridine kinase (EC 2.7.1.48) that catalyses the conversion of uridine into UMP, were not identified by sequence homology in *M. tuberculosis* genome (Cole et al. 1998).

Functional and structural studies of MtCDA enzyme were described previously (Sánchez-Quitian et al. 2010, 2015); however, no information about the direct essentiality of *cdd* gene and its role in *M. tuberculosis* infection is available. Here, we describe the construction of an *M. tuberculosis* knockout strain for *cdd* gene (KO), evaluation of mRNA expression of *cdd, deoA* and *add* genes, assessment of protein expression by MudPIT LC-MS/MS, *in vitro* growth studies, and analysis of *cdd* deletion in *M. tuberculosis* invasion and growth in a macrophage model of infection. To evaluate the effects of *cdd* disruption on *M. tuberculosis* growth, the KO strain was compared with *M. tuberculosis* H37Rv wild-type (WT) and complemented (CP) strains.

To construct the KO strain, a fragment of 1,788 bp containing the *cdd* gene (402 bp) with its flanking region (Fig. 1A) was amplified by polymerase chain reaction (PCR) from *. tuberculosis* H37Rv genomic DNA, using primers forward (5’-tttt**tctaga**cccagcgttgggcaacgaagt-3’) and reverse (5’-tttt**tctaga**gcaccctcagccagcttcttg-3’), both containing *XbaI* restriction sites (in bold). The 1,788 bp fragment was subsequently cloned into pUC19 using the *XbaI* restriction site. The *cdd* gene was disrupted by the insertion of a kanamycin cassette from pUC4K into unique internal enzyme restriction site *NotI* (Fig. 1B). Insert was released from pUC19 derivative vector by digestion with *XbaI,* and subcloned into *XbaI* linearized pPR27*xylE* vector (pPR27*xylE::cdd* kan) (Fig. 1B). The pPR27*xylE* plasmid contains a thermosensitive origin of replication, the *xylE* reporter gene, and the *sacB* counterselectable marker (Pelicic et al. 1997). *M. tuberculosis* H37Rv strain was transformed by electroporation with ~ 2 μg of pPR27*xylE*::*cdd* kan plasmid. Possible KO clones were selected in two steps, as described previously (Villela et al. 2017). Genomic DNA was isolated and PCRs were carried out using gene-specific screening primers forward (5’-gt- gtctttgcggctgtagtc-3’) and reverse (5’-gggcagttcatctcc- gtca-3’) to determine whether the WT or the KO strain was present in the targeted chromosomal region (Fig. 1B). Among the nine clones screened for the KO of *cdd* gene, all amplified a band of 3,293 bp compatible with a doublecrossover gene replacement event (Fig. 1C).

**Fig. 1:**
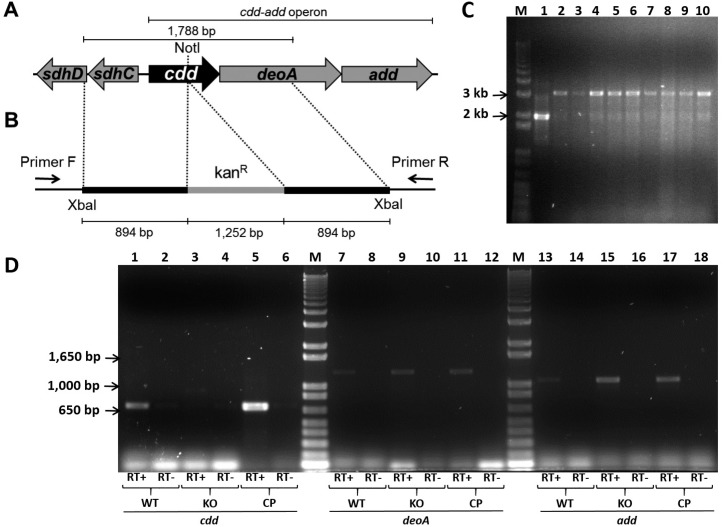
genomic environment of *cdd* gene in *Mycobacterium tuberculosis* (A), regions cloned into pPR27*xylE* vector (B), agarose gel electrophoresis of polymerase chain reaction (PCR) products from knockout clones (C), and mRNA expression of *cdd, deoA,* and *add* genes (D). (A) Genomic region of *cdd* gene (402 bp) containing unique internal *NotI* site and flanking genes; the operon *cdd-add* is indicated. (B) The *cdd* gene and flanking regions were amplified by PCR from *M. tuberculosis* H37Rv genomic DNA, and the *cdd* gene was disrupted by the insertion of a kanamycin cassette (kan^R^) into *NotI* site (cdd::kan^R^). The cdd::kan^R^ fragment was cloned into pPR27*xylE* vector using *XbaI* restriction site. Annealing regions of gene-specific screening primers forward (Primer F) and reverse (Primer R) for the possible knockout strains of *cdd* gene are indicated. (C) Agarose gel electrophoresis of PCR products from possible knockout clones. M - molecular marker 1 kb plus DNA Ladder (Invitrogen), PCRs were carried out with: Lane 1 -*M. tuberculosis* H37Rv genomic DNA, Lanes 2-10 - possible knockout clones genomic DNA. (D) mRNA expression of *cdd, deoA,* and *add* genes. M - molecular marker 1 kb plus DNA Ladder (Invitrogen). Lanes 1-6: *cdd* cDNA amplification (686 bp). Lanes 7-12: *deoA* cDNA amplification (1,284 bp). Lanes 13-18: *add* cDNA amplification (1,098 bp). cDNA synthesis from *M. tuberculosis* H37Rv (wild-type) was performed with reverse transcriptase enzyme (RT+) (lanes 1, 7, 13) or without (RT-) (lanes 2, 8, 14). cDNA synthesis from *cdd* knockout strain (KO) RT+ (lanes 3, 9, 15) and RT- (lanes 4, 10, 16). cDNA synthesis from complemented strain (CP) RT+ (lanes 5, 11, 17) and RT- (lanes 6, 12, 18).

To obtain the CP strain, a fragment containing the *cdd* gene, its upstream (183 bp) region containing the natural promoter, and 101 bp downstream to *cdd* was amplified by PCR from *M. tuberculosis* H37Rv genomic DNA using primers forward (5’-gggg**tctaga**ttgtcgccgttgtattcacc-3’) and reverse (5’-gggg**tctaga**gtcggtataggccttgacga-3’), both containing *XbaI* restriction sites (in bold). Next, the amplicon was cloned into *XbaI* linearised pNIP40/b(pNIP40::cdd), a mycobacteriophage Ms6-derived integrative vector (Freitas-Vieira et al. 1998), and the KO strain was transformed by electroporation with pNIP40::cdd, as described previously (Villela et al. 2017). The stability of the mutation introduced by gene replacement in *M. tuberculosis* was evaluated by plating KO and CP strains on media with and without antibiotics. The difference between the colonies obtained on plates containing antibiotics was not significant when compared with the ones obtained on plates without antibiotic, which indicates that the introduced mutation is stable (data not shown).

As mentioned previously, the *cdd* together with *deoA* and *add* genes are predicted to form an operon. Thus, to investigate the effect of *cdd* disruption on *deoA* and *add* genes, levels of mRNA expression of *cdd, deoA,* and *add* genes were monitored in WT, KO and CP strains. Ten milliliters of *M. tuberculosis* cultures were grown up to an OD_600_ of 0.6 - 0.8. The cell pellet was suspended in 1 mL of TRIzol (Invitrogen, Carlsbad, CA, USA), and disrupted in 2 mL screw-cap tubes containing 0.1 mm silica spheres using a L-Beader 3 (Loccus Biotecnologia, Cotia, Brazil). The aqueous phase was extracted with 200 μL of chloroform, and RNA was precipitated with 500 μL of isopropanol. Remaining DNA in RNA samples was digested with DNAse (RNAse free DNAse set, QIAgen, Hilden, Germany) and RNA samples were purified by using an RNA purification kit (RNeasy mini kit, QIAgen, Hilden, Germany). Synthesis of the first strand of cDNA was performed using 0.5 μg RNA from *M. tuberculosis* H37Rv, KO, and CP strains as template and random hex- amers as primers, following the instructions in the SuperScript III First-Strand Kit protocol (Invitrogen, Carlsbad, CA, USA). An aliquot of cDNA synthesis reaction was used to amplify *cdd* gene with primers *cdd* F (5’-ggggtc- tagattgtcgccgttgtattcacc-3’) and *cdd* R (5’-ggggtctagagtc- ggtataggccttgacga-3’), *deoA* gene with primers *deoA* F (5’- cgcatatgaccgacttcgcattcgacgcccc-3’) and *deoA* R (5’-agaagctttcagacgatccgatcgacgattagc-3’), and *add* gene with primers *add* F (5’-gtcatatgaccgctgcgccgaccctgcag-3’) and *add* R (5’- ctggatcctcactcgctgtgacccatgagc -3’). Amplification products were analysed in 1 % agarose gels. As shown in Fig. 1D, no *cdd* expression was observed in KO strain (lane 3), but was detected in WT (lane 1) and CP (lane 5) strains. The *deoA* and *add* mRNA expression were observed in all strains (Fig. 1D), which indicated the disruption of *cdd* by insertion of a kanamycin cassette did not exert a polar effect on the expression of these genes. No or minor DNA contamination was observed on negative controls, in which the cDNA synthesis was performed without the reverse transcriptase enzyme (Fig. 1D).

Although the transcription of *deoA* and *add* genes were not affected by *cdd* deletion in KO strain, the expression of downstream genes to *cdd* might be affected at the protein level if the translation is coupled. Coupled translation was observed in *Escherichia coli,* where the translation efficiency of one gene affects indirectly the translational level of downstream genes within an oper-on, potentially causing a strong phenotype (Levin-Karp et al. 2013). The fact that the start and stop codons of *cdd, deoA* and *add* genes overlap strengthens the possibility of a coupled expression. Therefore, Liquid Chromatography and Tandem Mass Spectrometry (LC-MS/MS) and Multidimentional Protein Identification Technology (MudPIT) analyses were performed to evaluate MtCDA, MtTP, and MtAD expression levels in WT, KO and CP strains. Cytoplasmic fractions of mycobacterial protein extracts from WT, KO and CP strains were obtained by ultracentrifugation, as described (Gunawardena et al. 2013). Chloroform/methanol protein precipitation was performed in WT, KO and CP cytoplasmic fraction (200 μg) according to (Wessel & Flügge 1984). Protein pellets were resuspended in 100 mM Tris HCl pH 8.5 containing 8 M urea, and digested according to Klammer and MacCoss (2006). Chromatographic separations were performed on a nanoLC Ultra 1D plus with autosampler (Sciex, Framingham, MA, USA) connected to a LTQ-XL Orbitrap Discovery hybrid instrument (Thermo Fisher Scientific, Waltham, MA, USA) through a nanoeletrospray ion source (Thermo Fisher Scientific, Waltham, MA, USA). Biphasic MudPIT columns and capillary analytical columns were prepared in house (Villela et al. 2015). MudPIT analysis was carried out according to Wolters (Wolters et al. 2001). Analyses were performed in technical triplicates. Data was collected with one MS1 full-scan in the Orbitrap (400-1600 m/z range; 30,000 resolution) followed by data dependent CID MS/MS spectra of the eight most intense ions in the ion trap, with dynamic exclusion applied. Mass spectra were searched for candidate peptides with the software Comet (Eng et al. 2012) in the platform PatternLab for Proteomics (Carvalho et al. 2016). The database contained a non-redundant *M. tuberculosis* reference proteome (ID UP000001584, www.uniprot.org) and the reverse sequences of all entries. The validity of the peptide spectra matches (PSMs) was assessed using the module Search Engine Processor (SEPro) from Patternlab for Proteomics, with a false discovery rate of 1%. Normalised spectral abundance factor (NSAF) was calculated according to Zybailov et al. (2006), and data were evaluated with the one-way ANOVA analysis, followed by Bonferroni's post-test, using GraphPad Prism 5.0. Differences were considered significant at the 95% level of confidence. With average outputs of 76,880 spectra and 12,700 unique peptides per MudPIT run, the proteomic pipeline applied here identified a total of 2075 mycobacterial proteins in the cytoplasmic fraction of the three strains, considering only proteins identified consistently in three technical replicates (data not shown). Spectra matching peptides from MtAD and MtTP were identified in WT, KO and CP protein extracts. A significant decrease in the level of MtTP (encoded by *deoA* gene) was observed in both KO and CP strains when comparing with WT strain (Fig. 2). This result suggests the occurrence of translational coupling between the neighboring genes *cdd* and *deoA.* On the other hand, the levels of MtAD, encoded by the third gene in the operon (*add*) (Fig. 1A), was not affected by the disruption of *cdd* gene (Fig. 2). This difference in translation interdependency might be explained by the fact that the disrupted *cdd* gene is closer to *deoA* than to *add* (Fig. 1A). MtCDA peptides were identified in both WT and CP but not in KO protein extracts (Fig. 2), indicating that the disruption of *cdd* gene abolishes the production of MtCDA protein in the KO strain. MudPIT LC-MS/MS results are in agreement with mRNA expression evaluation, in which *cdd* gene expression was identified in WT and CP strains but was absent in KO strain (Fig. 1D). MtCDA peptides were only detected in WT strain using ultracentrifugation for protein fractionation and MudPIT for peptide fractionation and identification. Seven spectral counts of two MtCDA unique peptides were identified in the WT strain technical triplicates. In the CP strain technical triplicates, 464 spectral counts of 10 MtCDA unique peptides were identified, a 66-fold difference in the levels of MtCDA in WT and CP strains. The difference in MtCDA levels between WT and CP strains could be explained by the fact that, though expressed by MtCDA natural promoter, the complemented copy of *cdd* gene is integrated in a different genome region.

**Fig. 2:**
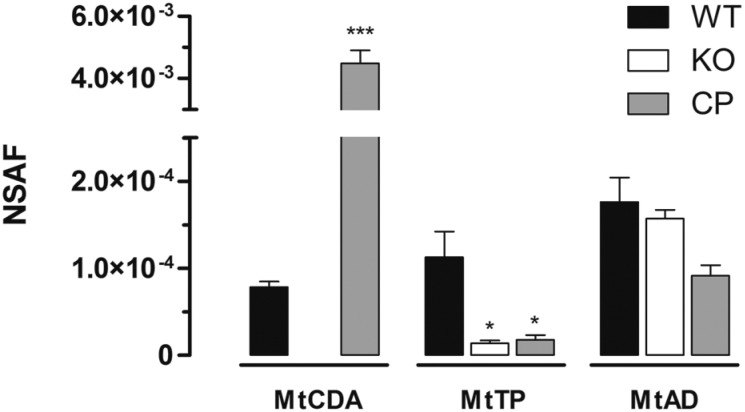
evaluation of MtCDA, MtTP and MtAD expression. MtCDA, MtTP and MtAD normalised spectral abundance factor (NSAF) in wild-type (WT), knockout strain (KO) and complemented strain (CP) cytoplasmic fractions identified by Multidimentional Protein Identification Technology (MudPIT). Bars represent average identification in technical triplicates. Asterisks represent significant differences between WT and KO or CP strains of each protein by one-way ANOVA analysis followed by Bonferroni post-test, *p < 0.05, ***p < 0.001.

The growth rate of the WT, KO, and CP strains were compared to determine whether *cdd* disruption leads to alterations during *in vitro* cultivation of *M. tuberculosis.* Growth curves were started at an optical density at 600 nm (OD_600_) of 0.01 in Middlebrook 7H9 10% OADC 0.05% tween-80 containing proper antibiotics, in triplicate, at 37°C, 80 rpm. Aliquots were removed from each culture at different time points and the OD_600_ was determined. As shown in Fig. 3A, the three strains have a similar pattern of growth when grown in Middlebrook 7H9 medium.

**Fig. 3:**
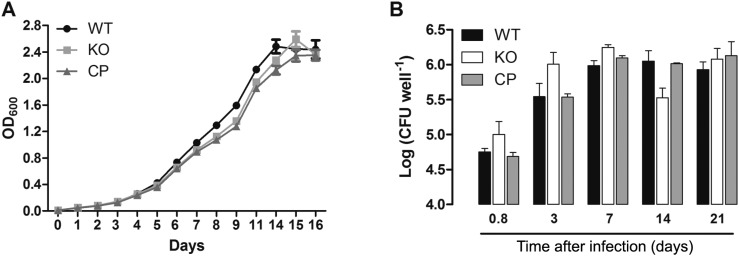
growth curve (A) and macrophage infection (B) with wild-type H37Rv (WT), knockout for *cdd* gene (KO), and complemented (CP) *Mycobacterium tuberculosis* strains. (A) Growth curves were started at an OD_600_ of 0.01 in Middlebrook 7H9 medium, in triplicate. (B) The macrophage infection results are expressed as mean numbers of the logarithms of colony-forming unit (CFU) per well of each strain of three independent measurements.

To examine whether the *cdd* gene was important for invasion and growth in phagocytic cells, we determined the bacterial loads of the WT, KO, and CP strains by using the macrophage model of infection. RAW 264.7 macrophage cell line was cultured and infected with WT, KO or CP *M. tuberculosis* strains as described previously (Villela et al. 2017), with minor modifications. Briefly, infection of macrophages was performed at a multiplicity of infection of 1:1 (bacteria/macrophage) at 37°C with 5% CO_2_. After 18 h, infection was terminated, and at this time point, 3, 7, 14 and 21 days of incubation, infected macrophages were lysed (Rodrigues-Junior et al. 2014), and plated on Middlebrook 7H10 agar supplemented with 10% OADC. Colony-forming unit (CFU) was evaluated after incubation of plates for three weeks at 37°C. This experiment was performed in triplicate, and the results were expressed as mean numbers of the logarithms of CFU per well, and were evaluated with the two-way ANOVa analysis, followed by Bonferroni's post-test, using GraphPad Prism 5.0. Differences were considered significant at the 95% level of confidence. As shown in Fig. 3B, no significant differences were observed in intracellular growth among WT, KO, and CP strains in macrophages after 18 h, three, seven and 21 days after infection. A decrease in bacterial load of KO strain was observed after 14 days of macrophage infection; however, compared to WT, it was not statistically significant (Fig. 3B). Although the decrease in bacterial load of KO strain after 14 days of macrophage infection was shown to be statistically significant (p < 0.05) when compared with CP strain, the analysis of the area under the curve revealed very similar intracellular bacterial growth for WT (total area mean ± standard error of the mean: 23.25 ± 0.47), KO (23.32 ± 0.27), or CP (23.06 ± 0.11) strains. Therefore, these data suggest that the disruption of *cdd* gene does not affect the *M. tuberculosis* growth in RAW 264.7 cells in the experimental conditions employed here.

In conclusion, a *M. tuberculosis* KO strain for *cdd* gene was constructed by allelic replacement. The CP strain was obtained by transforming the KO with pNIP40::cdd plasmid that expresses *cdd* gene from its natural promoter. The *cdd* deletion was validated at the RNA level, and was confirmed at the protein level by MudPIT LC-MS/MS. The disruption of *cdd* gene did not affect the mRNA expression of *deoA* and *add* genes, both located downstream of *cdd* on the same operon. However, MudPIT LC-MS/MS data indicated the MtTP protein level was decreased in both KO and CP strains, when compared with its level in WT strain, which could be explained by a translational coupling between *cdd* and *deoA* genes. The *M. tuberculosis* growth kinetics in Middlebrook 7H9 medium was not affected by the disruption of *cdd* gene. The results for RAW 264.7 cells suggest that the *cdd* gene product plays no role in *M. tuberculosis* invasion, growth and virulence in macrophages. Even though the recycling of nucleotides and nucleosides might represent a significant energy saving for the cell, the MtCDA activity is not required in the context of *in vitro* growth and macrophage invasion and infection by *M. tuberculosis* under the experimental conditions employed here. These findings could be explained by the redundancy found in nucleotide metabolism of *M. tuberculosis.* Although MtCDA is the only enzyme identified by sequence homology in *M. tuberculosis* nucleotide biosynthesis pathway that is able to rescue cytidine and deoxycytidine (Villela et al. 2011), there are enzymes from *de novo* pathway that synthesize all pyrimidine nucleotides from simple precursors (Warner et al. 2014). These enzymes might compensate for the absence of *cdd* gene in KO strain. However, it should be pointed out that studies under hypoxic and/or nutrient limitation conditions that likely mimic the environment in which latent bacilli survive should be pursued to provide a solid experimental basis for the role of *cdd* gene product. Different results of gene essentiality and importance for *M. tuberculosis* growth and survival might be obtained under different experimental conditions.
